# Unfolding neural diversity: how dynamic three-dimensional genome architecture regulates brain function and disease

**DOI:** 10.1038/s41380-025-03056-3

**Published:** 2025-05-23

**Authors:** Brandon L. Logeman, Steven F. Grieco, Todd C. Holmes, Xiangmin Xu

**Affiliations:** 1 Department of Molecular and Cellular Biology, Howard Hughes Medical Institute, Center for Brain Science, Harvard University, Cambridge, MA USA; 2 Department of Anatomy and Neurobiology, School of Medicine, University of California, Irvine, CA 92697 USA; 3 Center for Neural Circuit Mapping, University of California, Irvine, CA USA; 4 Department of Physiology and Biophysics, School of Medicine, University of California, Irvine, CA USA; 5 Department of Microbiology and Molecular Genetics, University of California, Irvine, CA USA; 6 Department of Biomedical Engineering, University of California, Irvine, CA USA; 7 Department of Computer Science, University of California, Irvine, CA USA

**Keywords:** Neuroscience, Psychiatric disorders

## Abstract

The advent of single cell multi-omic technologies has ushered in a revolution in how we study the impact of three-dimensional genome organization on brain cellular composition and function. Transcriptomic and epigenomic studies reveal enormous cellular diversity that is present in mammalian nervous systems, raising the question, “how does this diversity arise and for what is its use?” Advances in the field of three-dimensional nuclear architecture have illuminated our understanding of how genome folding gives rise to dynamic gene expression programs important in healthy brain function and in disease. In this review we highlight recent work defining how neuronal identity, maturation, and plasticity are shaped by genome architecture. We discuss how newly identified genetic variations influence genome architecture and contribute to the evolution of species-unique neuronal and behavioral functional traits. We include examples for both humans and model organisms in which maladaptive genomic architecture is a causal agent in disease. Finally, we make conclusions and address future perspectives of dynamic three-dimensional genome (4D nucelome) research.

## Introduction

The mammalian brain functions through the coordinated actions and interactions of millions-to-billions of cells, which orchestrate a wide range of organismal activities including physiological homeostasis, innate behaviors, learning and memory, and mood and cognition. To do so, the brain relies on thousands of transcriptionally distinct cell-types to execute coordinated functions that ultimately rely on specific and timely gene expression programs. These cell-types emerge as unique elements during development despite sharing a common genome, underscoring the importance of transcriptional control.

Fundamental understanding of transcriptional control originated from studies examining gene expression in single-celled organisms with compact genomes, and they showed the importance of control at transcriptional start sites. However, mammalian transcription has proven to be significantly more complex, with distal regulatory elements functioning as components governing gene expression in combinatorial fashion. The importance of distal regulatory elements in mammals is underscored by findings that the majority (~90%) of disease associated variants are located outside the coding portion of the affected gene.

Given the complexity of the mammalian nervous system, it is of little surprise that control of neuronal gene expression is distinctive in several aspects. One of these distinctions is that mammalian neurons express genes that are significantly larger than those expressed by non-neural tissues [1, 2]. This comes with costs -- the gene encoding the synaptic adhesion protein contactin-associated protein-like-2 (CNTNAP2) is over 2.3 Mb in length and requires upwards of 10 h to express a single transcript [3]. Additionally, genes expressed in neurons are controlled via 2-to 3-fold more regulatory elements (REs) than genes expressed in non-neuronal cells. Furthermore, these REs are distributed across genomic territories twice the size of those present in non-neuronal cells [4]. A recent genomic census of the mutational burden associated with neurodevelopmental disorders, such as intellectual disability and autism spectrum disorders (ASD), reveals that the genes associated with epigenetic and chromatin-related processes are more frequently mutated than the genes associated with synaptic processes [5].

To facilitate coordinated discovery of the structure and dynamics of mammalian genomes in time and space, the 4D Nucleome (4DN) network was established to pursue deeper knowledge of the biophysical and molecular factors that determine genome organization [6]. Phase one of the 4DN consortium was completed in 2020 with milestones including the development, validation, and benchmarking of several new technologies to aid in the understanding of genome function across time and space. In phase two, the 4DN network seeks to extend these findings to live cells, including primary neurons and intact brain tissue [7]. This concerted focus starts to provide a wealth of new insights into how the mammalian brain establishes and maintains the vast heterogeneity of cell-types that are necessary to execute the broad array of tasks required from nervous systems.

As part of the 4D Nucleome (4DN) consortium publication package, this review article introduces fundamental genomic architecture and organization principles and highlights recent studies on 4DN-related works in neuroscience.

## Principles of genomic architecture and organization

### The linear genome

Mammalian genomes exist as strands of deoxyribonucleic acids (DNA) wrapped around a histone octamer core in a complex referred to as chromatin. Classical models suggested that the genome is a linear polymer akin to a ticker tape in which transcriptional machinery would scan until it identified a designated binding site. Many functional features can be explained with this analogy. However, a more complete understanding of transcriptional control requires consideration of chromatin configuration across space (3D) and time (+1D). We begin by introducing the molecular features of the linear genome and then illustrate the need for 3D models to fully explain mechanisms of gene expression and cell-type diversity through time.

#### DNA methylation

**Fig. 1:**
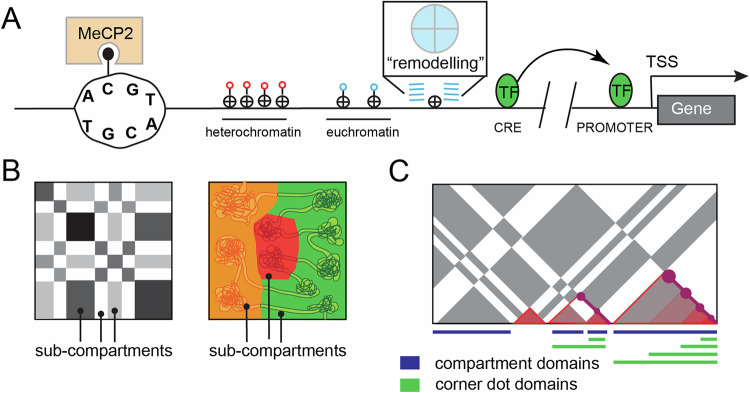
Characterizing the linear and 3D genome. Schematic illustrations of the linear and 3D genome structures. **A** A DNA strand is unwound revealing that only cytosine gets methylated and that MeCP2 acts as a “reader” by binding to methylated cytosines. A clutch of tightly spaced nucleosomes with red lollipops depicting heterochromatin and loosely spaced nucleosomes with blue lollipops depicting euchromatin. A nucleosome has an octameric core with 4 histone types that can be modified. Right next to this is a nucleosome that is in the process of being remodeled with a zoom in box depicting how chaperones/remodelers like nBAF can move things around to open up, close, or switch out nucleosomes. A CRE that is devoid of nucleosomes and occupied by TF(s), a break in the DNA to indicate distance, and a promoter devoid of nucleosomes and bound by a TF(s). **B,** **C** Newer high resolution chromatin contact maps show that a hierarchical model of TADs inside of compartments does not capture the characteristics of the 3D genomic structure -- there are compartments and sub-compartments as well as TADs and sub-TADs (conceptually re-created from Beagan et al., 2020) [58].

Of the four nucleic acids comprising DNA, cytosine is unique in its ability to support the covalent modification of a 5’ methyl group (Fig. 1A). A rich history of literature associates cytosine DNA methylation (5mC) with neuronal function, animal behavior, and various diseases [8]. Although 5mC predominantly occurs at CpG sites (mCG), with greater than 85% of all CpG sites methylated in adult brains, neurons but not glia acquire non-CpG cytosine methylation (mC**H**, where **H** can be A, C or T) at levels approaching that of mCG [9, 10]. Notably this acquisition of mC**H** occurs concurrently with synaptogenesis and is influenced by gene expression [11]. Both mCG and mC**H** modulate transcription factor (TF) binding and gene transcription through dynamic interactions at regulatory elements and gene bodies [12]. Both types of methylation directly influence the DNA binding of methyl-CpG binding protein 2 (MeCP2), a crucial 5mC reader that when malfunctioning, is a cause of Rett syndrome [10, 13, 14]. Genome-wide differential methylation analyses predict millions of regulatory elements and have produced a cellular taxonomy and a single base-resolution genome atlas.

#### Nucleosomes

To package and organize the genome within the nucleus, DNA is compacted by being wrapped around a highly dynamic core of histone proteins to create the fundamental unit of chromatin known as the nucleosome (Fig. 1A). Each nucleosome is comprised of two copies of each of the four core histones (H2A, H2B, H3 and H4) with ∼147 bp of DNA [15]. Histone proteins possess a flexible tail which protrudes from the DNA wrapped core and is amenable to a variety of post translational modifications that greatly affect protein recruitment and transcriptional activity [16]. Histones are governed by dedicated chaperone and remodeling complexes that endow specific regions of chromatin with temporally-precise functions [17].

Nucleosome remodelers, a large family of ATP-dependent protein complexes that alter nucleosome structure and regulate chromatin accessibility, are particularly important in the nervous system. Pathogenic mutations in remodelers are over-represented in human neurodevelopmental and psychiatric disorders, including ASDs, intellectual disabilities, epilepsy and schizophrenia (SCZ) [18]. While much of the remodeling machinery is ubiquitously present in all cells, neurons also possess a unique complex known as neuro-specific BAF (nBAF). Patients with mutations that destabilize nBAF present with ASD and show reduced cerebral white matter volume in addition to corpus callosal hypoplasia. Mouse models lacking nBAF across multiple genetic backgrounds display reduced social interaction and synaptic plasticity in addition to hyperactivity and memory-impairment. These phenotypes are potentially mediated by altered activity-responsive transcriptional programs and highlight the unique requirements of neural-gene expression programs.

#### *Cis* regulatory elements

Mammalian gene expression is influenced by *cis* regulatory elements (CREs). CREs control spatiotemporal gene expression through the binding of sequence-specific transcription factors (TFs) and through the recruitment of chromatin remodeling proteins and/or transcription machinery (Fig. 1A). These elements, including promoters, enhancers, insulators, repressors and other less-well-characterized regulatory sequences, strongly correlate with hypomethylated regions of DNA. They work together to drive cell-type-specific gene expression during animal development, cell differentiation, and in disease states.

In contrast to the ~20,000 protein coding genes, the adult mouse brain possesses ~1 million CREs that comprise ~20% of the genome [19]. CREs tend to feature cell-type-restricted accessibility, with ~25% CREs active in less than 1% of all cell types. Further highlighting the unique complexity governing neuronal gene expression through CREs is the observation that each gene is influenced by a median of 24 putative enhancers, representing a 2- to 3-fold increase relative to non-neuronal tissue. The majority of these enhancers is located in intergenic regions or in the introns of gene bodies, with the median distance between CRE and transcriptional start site being 156 kb.

The mechanisms through which promoters stimulate transcription can be readily described though one-dimensional models of the genome. Sequence-specific transcription factors bind proximal to the transcriptional start site and recruit expression machinery to begin local transcription. In contrast, distal elements, such as enhancers and repressors, act in concert with promoters to modulate transcription over significant distances and often skip nearby promoters non-linearly. These observations, together with recent studies that strongly suggest enriched physical proximity between cognate CRE–promoter pairs, necessitate the importance of examining the genome in three dimensions [20].

### The 3D genome across time

For CREs to effectively interact with their target promoters, they must be brought into close physical proximity. In recent years, substantial progress has been made toward understanding the forces governing such chromatin organization [21–24]. These studies reveal two separate and competing mechanisms that dynamically give rise to genome folding (Fig. 1B, C). We describe these two forces and highlight examples in which their disruption leads to profound neurological defects.

#### Compartment domains

Early microscopy experiments utilizing differential DNA staining techniques recognized that nuclear DNA exists as two separate forms: euchromatin and heterochromatin [25]. Extensive studies have since realized that euchromatin is characterized by the presence of actively transcribed genes, wider spacing between nucleosomes, higher accessibility, and histone modifications such as H3K4me3, H3K27ac, H4K8ac, and H4K16ac, as well as histone variants H2A.Z and H3.3 [26, 27]. By contrast, heterochromatin is transcriptionally inactive, less accessible, and is decorated with H3K9me3 and H3K27me3 histone marks [26, 27]. Recent chromosome conformation techniques have shown these two types of chromatin alternate along the length of a chromosome and preferentially interact with distal regions between the same types [28–33]. Chromatin contacts are sufficient to identify these two states: euchromatin are referred to as the ‘A’ (active) compartment and heterochromatin referred to as the ‘B’ (inactive) compartment [34, 35] (Fig. 1B). Further studies show that within A and B compartments, there exists sub compartments with specific combinations of epigenetic marks that correspond to nuclear localization [36–39]. Portions of the A compartment are localized near nuclear speckles, while distinct components of the B compartment are localized as lamina-associating domain (LADs) [40] and the nucleolus-associating domains (NADs) [41].

In most non-neuronal cell types these compartment domains exhibit a clear spatial segregation. The heterochromatin are mainly localized at the nuclear periphery and the region surrounding the nucleoli, while euchromatin is positioned in the interior of the nucleus [42]. Neurons possess comparatively weak spatial localization of chromatin compartments. Localization of genes to LADs tends to repress transcription, however in neurons LADs are not that repressive and a greater percentage of heterochromatin is found near the nuclear interior [43, 44]. Perhaps the most striking example of an aberration of this structure is revealed in patients with Hutchinson-Gilford progeria syndrome, a disease caused by mutations in the genes of the major components of the nuclear lamina resulting in severe changes in nuclear organization and nuclear compartmentalization. Although Hutchinson-Gilford progeria syndrome presents with systemic and dramatic aging, cogitative processes remain largely unimpaired and highlight the uniqueness of the neuronal nucleus [45].

While the presence of A and B compartments is now well-established, the mechanisms that drive the organization of these compartments remains an active area of research. One leading hypothesis suggests that liquid-liquid phase separation may partition heterochromatin from euchromatin – in other words euchromatin and heterochromatin are like oil and water and just separate [46–48]. Given that some chromatin-associated proteins show properties of phase separation, this presents an attractive model for segregation of active and inactive chromatin [49, 50]. HP1-alpha and the Polycomb complex, which interact with heterochromatin via binding to H3K9me and H3K27me3 modified nucleosomes respectively, drive formation of droplets in vitro [46, 51]. Additionally, stretches of chromatin alone have been shown to form phase-separated droplets in a manner that is inhibited by p300-mediated acetylation of histone tails. Phase separation is rescued by addition of the H3K27ac binding euchromatin associated protein BRD4 [52]. In addition to BRD4, the euchromatin associated proteins FUS and TAF15 are also capable of forming microphase separated bodies, suggesting that the formation of both A and B compartments may be maintained though phase separation [52, 53]. However, concerns have been raised that other mechanisms may explain these observations and highlight the ongoing need for the development of robust in vivo perturbations [54, 55].

#### Loop domains

In addition to A/B compartment domains which interact with each other at similar frequences, the advent of chromatin-contact technologies revealed the presence of genomic blocks in which DNA sequences exhibit significantly higher interaction frequency within the domain than outside domain [56, 57]. There is substantial variation in the nomenclature used to describe these domains. Initially, low-resolution studies referred to these as Topologically Associating Domains (TADs) and suggested that they are invariant among cell-types and nested inside larger compartment domains. However, more recent ultra-resolution studies show sub-compartment domains [36, 38] consisting of multiple sub-TAD structures that appear to vary across cell-types and cell-states. Terminology to describe these structures continues to evolve [58], but here we refer to them broadly as ‘loop domains’ (Fig. 1C). Importantly, live-cell imaging experiments reveal that loop domains are highly dynamic, highlighting the notion that these domains represent an ensemble average interaction frequency across millions of cells [59, 60]. Single-cell Hi-C [61, 62] as well as super-resolution microscopy [63] experiments show that loop domain-like structures behave in single cells in a manner that is consistent with the loop extrusion process. Loop domains likely create insulated neighborhoods that demarcate enhancer search space for target genes -- locally co-regulated genes are frequently found together within the same loop domain potentially to make this search more efficient [64–66].

Progress has been made toward understanding the mechanisms that generate loop domains, and it is now widely regarded to be the result of ATP-dependent DNA extrusion bounded by architectural proteins. This is accomplished via the molecular motor complex cohesin, which binds DNA and tracks along the sequence thereby ‘extruding’ the intervening DNA and the DNA binding protein CCCTC-binding factor (CTCF) in the process. Cohesin extrusion proceeds along DNA until the complex encounters convergently oriented CTCF, which causes cohesin to stall and eventually dissociate. Direct evidence supporting this model is evident from in vitro single-molecule imaging studies in which cohesin translocates along naked DNA [67–69]. Acute depletion of cohesin activity results in a near complete loss of loop domains across multiple cell-types -- while also strengthening compartment domains [70–72]. These data suggest that loop extrusion and compartmentalization are distinct and competing forces and highlight the differences between these two mechanisms.

Although disruption of loop domains results in only modest changes in gene expression in non-neuronal cells [70, 73, 74], neurons are particularly sensitive to disruption in loop domains. Primary post-mitotic cortical neurons that are acutely depleted of cohesin display significant changes in gene expression, particularly in genes that typically show high contact probability with distal enhancers [75]. One possible explanation for this is that disruption of loop extrusion affects tissue-specific inducible genes that may be dependent on distal enhancer regulation, while expression of promoter-centric ‘housekeeping’ genes may not be affected as much by loop disruption [70].

Conceptually groundbreaking work examining gene expression from the family of ~60 clustered Protocadherins has revealed that the brain fine tunes cohesion activity to enable cell-type specific expression [76–78]. For instance, variation in cohesion processivity through modulation of the auxiliary protein WAPL results in differential promoter choice between serotonergic neurons, cortical neurons, and olfactory sensory neurons. One hypothesis for this neuron enriched importance comes from the understanding that while many cells are continually dividing and thus dependent on cohesion activity for successful DNA segregation, neurons are unique in that as G0 arrested cells, they are able to uncouple cell replication from the gene expression activity of cohesion.

Highlighting the importance of proper cohesion tunning and Protocadherin expression are the human genetic disorders Cornelia de Lange Syndrome (OMIM 122470) and CTCF-related disorder (ORPHA:363611). These conditions are caused by mutations that affect cohesion processivity, ultimately manifesting in neurodevelopmental disorders that present with intellectual disability [79, 80]. Cells derived from both patients [81] and mouse models [82, 83] of Cornelia de Lange Syndrome display altered Protocadherin expression, likely giving rise to the observed deficits in neural connectivity throughout development. Finally, additional neurodevelopmental and intellectual disorders that have not been formally diagnosed have been found to harbor genetic variants of the cohesion auxiliary protein WAPL [84], further supporting a model whereby cohesion activity plays a central role in brain wiring.

## Significance of genome structure and dynamics in neuroscience

### Cell-type classification

The diversity of brain cell types is maintained by regulatory mechanisms and gene expression patterns, including the 3D organization of the genome. Even relatively simple interrogation of genome arrangement by staining with dyes that bind DNA provides cell type categorization [85, 86]. This is because heterochromatin is more tightly compacted than euchromatin, staining of heterochromatin results in a brighter signal that is sufficient for identifying different cell types. More recent approaches, such as calculation of chromatin contact matrices from individual nuclei and determination of genome-wide compartmental assignment, more robustly allows for cell type classification [87, 88].

**Fig. 2:**
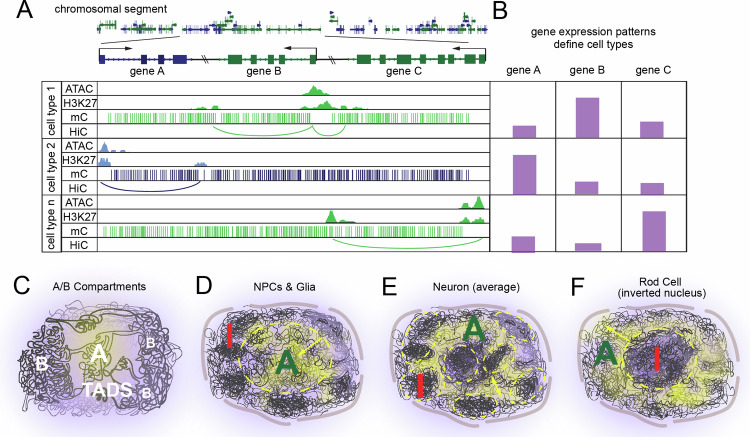
Defining cell types based on chromatin structure. Schematic illustrations of the role of 4D nucleomics in defining cell types. **A,** **B** A combination of 4DN-type techniques is comparable in resolution with transcriptomics to define cell types. **A** Genome browser tracks for 3 cell types depicting ATAC, H3K27ac, DNA methylation, and HiC loops. **B** A cell type gene expression matrix for 3 cell types and 3 genes highlighting how cell types are defined by 4DN characteristics. **C**–**F** Neural precursors and glia (and most cells in the body) predominantly show inactive (I, red) chromatin localized at the nuclear exterior while active (A, green) chromatin is located centrally. Neurons do not seem to follow this rule as strictly, with the extreme example of nocturnal photoreceptors being complexly inverted.

Recently cell census projects have employed single cell or single nucleus RNA sequencing as a method-of-choice to define cell types, due in part to the maturity and interpretability of this technique (Fig. 2A, B). Current efforts have resulted in a mouse brain cell atlas hierarchically organized into 4 nested levels of classification: 34 classes, 338 subclasses, 1201 supertypes and 5322 clusters [89, 90]. Impressively, incorporation of biochemical methods capable of identifying DNA methylation, chromatin accessibility, and chromatin contacts at the genome-wide scale provides a nearly 1:1 alignment with transcriptionally defined cell types, thus highlighting the role of genome organization in transcriptional regulation and cell type assignment [19, 91, 92].

One particularly striking example of neuronally-unique nuclear organization occurs in rod photoreceptor cells from multiple species of nocturnal animals [93] (Fig. 2C). Although they possess normal chromosomal looping and compartmentalization, these species lack expression of multiple components of lamin associated domains (LADs) [94–96] allowing for the strong forces mediating B component interactions to drive heterochromatin localization to the interior rather than periphery of the nucleus [97]. Due to differences in the refractive index between heterochromatin and euchromatin, this adaptation has been proposed to enable nuclei to act as collecting lenses to capture and focus low-level light, which is important for night vision. Intriguingly, a similar phenomenon of inverted topology occurs in mouse olfactory sensory neurons and implies that this method of genome arrangement may be implored for purposes beyond low light environments [98, 99]. Specifically, this “inside-out” topology may be advantageous for stochastic regulation of olfactory receptor gene choice, as disruption of this nuclear topology results in aberrant expression of olfactory receptor genes [98].

### Development

**Fig. 3:**
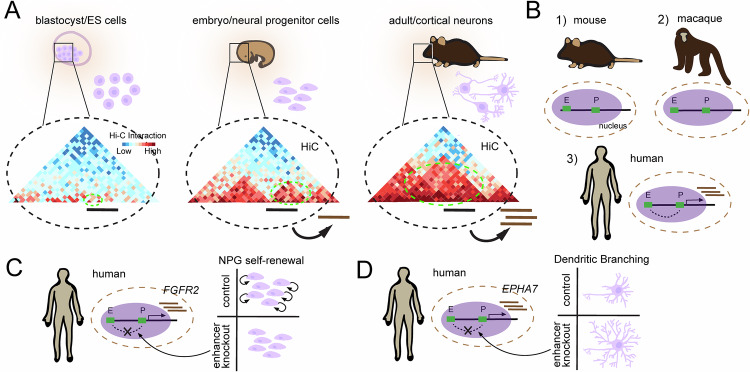
Development of characteristics based on chromatin structure. Schematic illustrations of the role of 4D nucleomics in the development of species characteristics. **A** Depiction of the three stages of development which contain blastocyst/ES cells, embryo/neural progenitor cells, and adult/cortical neurons and the trend that differentiation induces novel enhancer-promoter (EP) contacts as determined with Hi-C data (conceptually re-created from Bonev et al., 2017) [102]. **B** Cartoon showing that there exists EP contacts that are unique to humans (conceptually re-created from Luo et al., 2021) [109]. **C** A human specific E-P loop that stimulates expression of FGFR2. Disruption of this human specific enhancer results in decreased self-renewal of neural progenitor cells. **D** A human specific enhancer that stimulates expression of EPHA7 -- deletion of this enhancer results in aberrant dendrite development (conceptually re-created from de la Torre-Ubieta L et al., 2018) [107].

During embryonic development, the appearance of the neural tube marks the first formation of what will become the central nervous system (CNS). Lining this structure are neural precursor cells (NPGs), and multipotent stem cells (MSCs) that are capable of differentiating into the major cell types of the CNS, including neurons, astrocytes and oligodendrocytes. Studies using ultradeep Hi-C with resolutions up to 750 bp that examine mouse and human neural differentiation both in vitro and in vivo, have begun to shed light on the nuclear reorganization associated with neuronal birth (Fig. 3A). Notably, stem cells exhibit a chromatin state that is globally more accessible than fully differentiated neurons [100, 101]. During lineage commitment an increase in interaction between B compartments is observed and represents the formation of heterochromatin [102]. Additionally, a substantial amount of the genome undergoes compartment switching with genes that change from compartment A to B showing reduced expression while genes that change from B to A show higher expression [103]. However, compartment switching is not required for gene activation. A more faithful predictor of gene expression is the presence or absence of dynamic enhancer-promoter (E-P) loops. A substantial number of E-P loops are either gained or lost during differentiation -- the emergence of E-P loops occurs concomitantly with gene expression. This supports a model where dynamic chromatin looping from enhancer regions triggers gene activation and shows a strong correlation between gene expression and developmental status [104]. An additional feature of genes that are *not* actively transcribed during development, is that they accumulate mC**H** that recruit MeCP2 to fine tune transcription later in life [11, 12].

Beyond model organisms, the study of comparative genetics has proven to be a driver of discovery for finding active elements of chromatin that influence the brain. Notably, humans have a significant expansion of cortical neurons compared to other mammals (Fig. 3B). This is hypothesized to involve an increase in cortical NPG proliferation that enables the formation of a greater diversity and number of cortical neurons [105]. The use of Hi-C and ATACseq datasets for probing the developing brains of humans, macaques, and mice provides a powerful approach for identifying functional genome configurations that relate to human-specific developmental phenotypes. FGFR2, encoding a fibroblast growth factor receptor necessary for cortical NPG self-renewal [106], was found to form a human specific loop ~550 kb upstream of its TSS (Fig. 3C). CRISPR-mediated disruption of this enhancer reduces FGFR2 expression in primary human NPGs by ~40% and decreases progenitor self-renewal [107]. Additionally, a human-specific E-P loop was discovered affecting EPHA7, a gene encoding a plasma membrane receptor for the signaling molecule ephrin shown to be involved in dendritic maturation [108, 109] (Fig. 3D). A luciferase reporter assay shows that the activity of this human enhancer is significantly higher than that of chimpanzee and macaque. Sequence alignment between multiple species reveals the creation of multiple TF binding sites unique to the human sequence. CRISPR-mediated disruption of this enhancer yields a ~ 50% reduction in expression in neurons differentiated from human NPGs and an increase in dendritic length, similar to the phenotype observed from an EPHA7 knockout.

### Plasticity

**Fig. 4:**
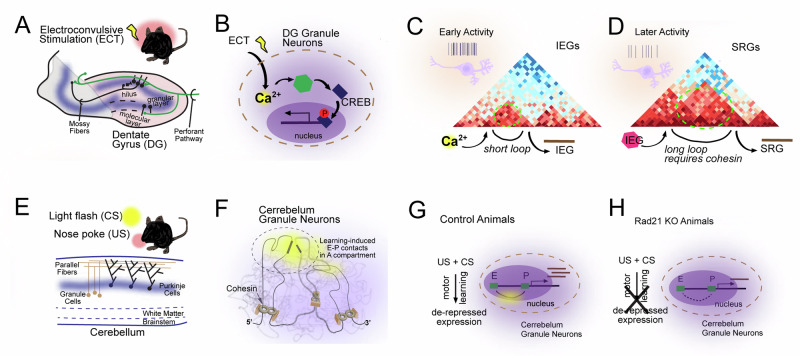
Role of chromatin structure and gene regulation in regulating neural plasticity. Schematic illustrations of the role of 4D nucleomics in neural plasticity. **A,** **B** Electroconvulsive stimulation (ECT) induces dentate gyrus (DG) granule neuron activity in hippocampus. **B** ECT-induced granule neuron activity induces transcription factor activity (CREB) leading to transcription of immediate early genes (IEGs) -- IRGs then induce secondary responses genes (SRGs) (conceptually re-created from Su et al., 2017) [110]. **C, D** The enhancer-promoter landscape of activity-induced genes can correlate with induction time. **E,** **F** Delay tactile startle conditioning used as a motor learning paradigm depends on granule cells of the cerebellum (conceptually re-created from Yamada et al., 2019) [118]. **F**–**H** Nose pokes learned to be associated with condition stimuli requires topological association domain (TAD) formation and other chromatin contacts dependent on the cohesin subunit Rad21.

Experience-dependent neuronal activity rapidly and robustly induces multiple gene expression programs that facilitate new protein synthesis necessary for the long-term retention of memories and the acquisition of learned skills. These longstanding ideas have been extended to epigenetic modulation following neural activity changes. Recent work has shown that synchronous neural activation of mouse dentate gyrus (DG) via electroconvulsive stimulation dramatically alters chromatin accessibility to facilitate gene expression [110] (Fig. 4A, B). This remodeling is initiated via a depolarization-induced influx of cytosolic calcium, which activates the CaM kinase family members which then phosphorylate the transcription factor CREB along with additional potential unknown targets. Phosphorylated CREB binds promoters of activity-regulated genes and recruits epigenetic modifying enzymes such as the histone acetyltransferase CBP, which stimulates the deposition of H3K27ac and further activates expression of activity-regulated genes. The nucleosome remodeling complex nBAF is also phosphorylated in a calcium-dependent manner, which decreases its interaction with the NuRD repressor complex, relieving inhibition and allowing it to bind H3K27ac, which further increases activity-regulated gene expression [111, 112].

The complexity of the enhancer-promoter landscape of activity-induced genes appears to correlate with induction time (Fig. 4C, D). Immediate early genes (IEGs) that are expressed within seconds to minutes of neuronal activity and require no new protein synthesis, tend to have relatively short dynamic enhancer-promoter (E-P) loops compared to secondary-response genes (SRGs), which are dependent on protein-synthesis and thus take longer operative timescales (hours) to be expressed [113]. Furthermore, IEG expression appears to be insensitive to cohesin depletion, perhaps due to the short-range nature of the loops employed to activate transcription [114]. This stands in contrast to findings that SRGs typically require cohesin to form the necessary E-P loops that underlie activation. This finding hints that fast, short-term gene expression may occur primarily through promoter action, but more dramatic gene expression programs may require complex E-P loops. This raises the question as to how the de novo formed E-P loops of IEGs are initialized in the absence of loop extrusion.

One intriguing possibility has been postulated as a result of the observation that neuronal activity induces DNA double strand breaks at the promoters of IEGs [115]. These breaks could immediately relieve genes from loop-mediated insulation and allow access to distal enhancers without the requirement of loop extrusion. Intriguingly, repair of double strand breaks requires the neuron-enriched histone variant H2A.Z [116] which has also been shown to be responsible for inactivating neuronal IEGs [117] More work will be required to investigate the nature of these activity-induced double strand breaks, and if they are adaptive.

Disrupting this activity-induced chromatin reorganization during learning processes might be predicted to inhibit memory formation. One example highlighting the requirement of activity-dependent chromatin reorganization for plasticity, is a motor learning paradigm that requires granule neurons in the cerebellum to undergo changes in E-P interactions to orchestrate the expression of gene programs associated with motor learning (Fig. 4E, F). Intriguingly, cell-type specific knockout of cohesin activity in these granule neurons disrupts the expression of learning associated gene modules as well as their combined E-P interactions, resulting in diminished learning and demonstrates a required in vivo, cell-type specific role for loop extrusion in motor learning [118].

### Evolution

**Fig. 5:**
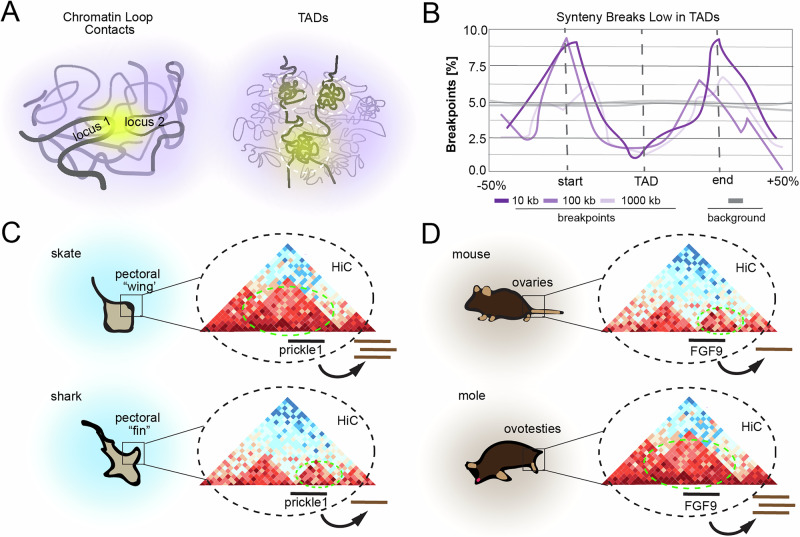
Evolution of characteristics based on chromatin structure. Schematic illustrations of the role of 4D nucleomics in the evolution of species characteristics. **A** Cartoon depicting chromatin loop contacts and toplogically associated domains (TADs). **B** Cartoon graph showing that synteny breaks are enriched at TAD boundaries and selected against inside of TADs. **C** A cartoon showing TAD rearrangement in skates/sharks leads to increased prickle1 expression (conceptually re-created from Marletaz et al., 2023) [123]), and (**D**) a cartoon showing that TAD rearrangement in mice/moles results in masculinization (conceptually re-created from Real et al., 2020 [126]).

3D genomic arrangement impacts evolutionary history. Across mammals, chromosomal rearrangements are enriched at TAD boundaries while being depleted inside TAD bodies [119, 120] (Fig. 5A, B). Expression of orthologous genes between human and mouse across 19 matched tissues shows higher correlation between genes that are present in conserved TADs compared to those that are in disrupted TADs. Thus, it may be advantageous to preserve certain TADs through evolution. Loop extrusion itself may at times introduce aberrant chromosomal rearrangements that affect 3D genomic arrangement, as chromatin is vulnerable to DNA double strand breaks because of mechanical stress incurred during loop extrusion [121]. However, multiple studies have shown that although individual TADs appear to be constrained, they are not invariant across deep evolutionary timescales. Novel E-P contacts created through TAD shuffling may provide selection advantage.

A notable example of how 3D genome topology rearrangement can influence animal behavior is seen by comparing two related species of cartilaginous fish: sharks and skates (Fig. 5C). Sharks have elongated, triangular pectoral fins, while skates have pectoral fins that extend forward and fuse with the head to form wing-like structures, channeling the ideas first put forth by D’arcy Thompson [122]. These distinctive anatomies enable skates and sharks to exhibit unique behavioral patterns. Hi-C analysis comparing skate, mouse, and gar revealed both conserved and unique TADs, highlighting that while regulatory constraints may influence genome structure, genomic topology is not invariant across deep evolutionary timescales [123]. Intriguingly, TADs unique to skates contain multiple genes involved in the WNT / PCP signaling pathway, known to regulate appendage growth in multiple species, and show increased expression of the key signaling gene prickle1. Furthermore, treatment with a Rho-kinase inhibitor, which disrupts WNT / PCP signaling, leads to less pronounced anterior expansion of the pectoral fin. Taken together, these findings suggest that TAD rearrangements had a role in recruiting and repurposing genes and pathways during the evolution of the unique batoid fin morphology.

Additional evidence supporting the role of 3D genome topology rearrangement in gene expression is found in multiple related species of moles in which females produce levels of testosterone similar to males [124] and develop prominent aggressive behavior [125] (Fig. 5D). The increased androgen levels are due to the presence of a hybrid reproductive organ known as an ovotestes, which contains a functional ovarian part that fully supports sexual reproduction and a testicular part that lacks fertile germ cells but contains cell populations more typically found in males, such as androgen-producing Leydig cells. Hi-C analysis of mole ovotestes identified a synteny break occurring in a TAD containing FGF9, a gene known to be involved in testes development. This inversion led to introduction of new regions of DNA marked by active epigenetic marks into the FGF9 TAD. While FGF9 expression is silenced in the developing gonad of most XX-genotyped mammals, moles showed sustained expression. Although the exact enhancers regulating this effect remain to be identified, overexpression of FGF9 in mice results in female-to-male sex reversal. This suggests that the novel TAD architecture of the mole genome modulates FGF9 expression in females, resulting in masculinized traits [126].

### Disease

In developed nations a substantial fraction of disease burden has a genetic underpinning, often relating to the inappropriate control of gene expression. By unfolding the genomic topology involved in regulating gene expression, 4DN research is well-suited for advancing our understanding of human disease. Here we highlight an area of active research in which 4DN studies are contributing to our better understanding of Alzheimer’s disease (AD).

AD is the most common cause of age-related dementia and impacts 50–100 million people worldwide. AD is neuropathologically characterized by abnormal protein aggregation (β-amyloid deposits and neurofibrillary tangles) and neuroinflammation, which correlates with brain atrophy and cognitive decline. There is emerging evidence that the immune system, particularly via the actions of microglia, contribute to AD progression. Notably, multiple recent AD GWAS studies have identified numerous variants that are associated with an increased risk of developing AD. Further epigenomic analysis shows that some of these loci are preferentially enriched in enhancer sequences that are implicated in innate immune processes [127].

**Fig. 6:**
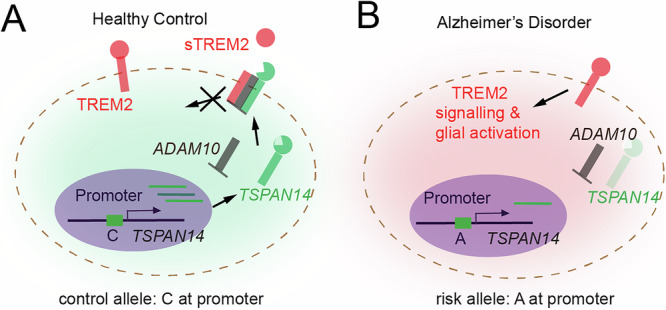
Defining disease based on chromatin structure. Schematic illustrations of the role of 4D nucleomics in defining diseases. **A,** **B** This depicts a GWAS identified variant associated with AD that is a Hi-C verified enhancer to the TSPAN14 gene, with the risk variant exhibiting less open chromatin at the enhancer and lower gene expression. This gene product assists with proper trafficking of the protease ADAM10 to the cell surface, where it cleaves the extracellular domain of TREM2, which is the second highest hit in late onset AD screens (conceptually re-created from Yang et al., 2023) [128].

Microglia play a key role in AD progression by triggering myeloid cell receptors (TREM2), a cell surface receptor of the immunoglobulin superfamily that upon ligand binding initiates a signaling cascade that promotes microglia activation (Fig. 6A). Intriguingly, the extracellular binding domain of TREM2 undergoes proteolytic cleavage by the family of ADAM proteases, resulting in a soluble form (sTREM2). ADAM10 proteases are trafficked to the cell surface via TSPAN14 where they are then able to cleave TREM2 (and also amyloid precursor protein (APP), releasing a neuroprotective cleavage product). Hi-C analysis reveals direct interaction between a top GWAS hit and the promoter of TSPAN14 in activated microglia. CRISPRi and base editing experiments confirm that this risk allele results in reduced expression of TSPAN14 and thus decreases cell-surface TREM2 ectodomain shedding by ADAM10 (Fig. 6B) [128].

### Conclusions and Future Perspectives

Recent advances have revealed that dynamic genome folding plays a crucial role in defining neuronal identity, development, and activity across an organism’s lifetime and even throughout evolutionary timescales. The 4D Nucleome onsortium and framework has been instrumental in uncovering these insights, providing powerful tools to manipulate chromatin structures in living systems. Given the vast cellular heterogeneity of the mammalian nervous system, a key challenge moving forward will be to understand how genome architecture varies across different neural cell types and how pathological mutations can cause cell-type-specific effects. Most research to date has focused on genetically identical mouse strains or human cell lines, but incorporating genetically diverse samples will be essential for identifying mechanisms that are consistent across populations. Additionally, since neurons are long-lived cells, understanding the maintenance of proper genome folding across decades of life will be critical, particularly in understanding neurodegenerative processes [129]. A glimpse of possible mechanism comes from the recent observation that olfactory sensory neurons employee solid phase separation, as opposed to the widely accepted liquid-phase separation, to ensure singular OR transcription for the life of the neurons [130]. Further studies will be required to determine if this can be considered a universal solution. Finally, the ability to manipulate 4DN structures offers exciting therapeutic potential to reverse or mitigate disease-causing chromatin aberrations, making it a vital focus for future neuroscience research. Overall, advancing our understanding of 4D genome dynamics in the nervous system, particularly across diverse cell types and genetic backgrounds, holds the promise of uncovering novel mechanisms of neural function and disease, with significant implications for developing targeted therapeutic strategies.
